# Mixed frequency composite indicators for measuring public sentiment in the EU

**DOI:** 10.1007/s11135-022-01468-9

**Published:** 2022-07-01

**Authors:** Raffaele Mattera, Michelangelo Misuraca, Maria Spano, Germana Scepi

**Affiliations:** 1 Department of Social and Economic Sciences, Sapienza University of Rome, Rome, Italy; 2 Department of Economics and Statistics, University of Naples “Federico II”, Naples, Italy; 3 Department of Business Administration and Law, University of Calabria, Rende, Italy

**Keywords:** Confidence indicator, Composite indicator, Public opinion, Media sentiment, Mixed data sampling

## Abstract

Monitoring the state of the economy in a short time is a crucial aspect for designing appropriate and timely policy responses in the presence of shocks and crises. Short-term confidence indicators can help policymakers in evaluating both the effect of policies and the economic activity condition. The indicator commonly used in the EU to evaluate the public opinion orientation is the Economic Sentiment Indicator (ESI). Nevertheless, the ESI shows some drawbacks, particularly in the adopted weighting scheme that is static and not country-specific. This paper proposes an approach to construct novel composite confidence indicators, focusing on both the weights and the information set to use. We evaluate these indicators by studying their response to the policies introduced to contain the COVID-19 pandemic in some selected EU countries. Furthermore, we carry out an experimental study where the proposed indicators are used to forecast economic activity.

## Introduction

Several studies documented, after Page and Shapiro (1983), the existence of a relationship between public opinion orientation, intended both as households and firms viewpoint, and public policy (e.g. Burstein 2003; Wlezien and Soroka 2007). As stated by Burstein (2003) *“No one believes that public opinion always determines public policy, few believe it never does”*. Previous evidence suggested that politicians react to public opinion pressure by introducing policies aiming at improving people and firms’ confidence about economic growth and, consequently, about the government’s action. On the other side, economic agents also adjust their behaviours based on public policies. Correctly measuring the sentiment is of extreme importance for policymakers to short-time track the state of the economy. This latter aspect relies on the fact that people and firms adjust their actions according to the expectations for future. The sentiment of households is usually viewed from a consumer perspective, considering the future developments of consumption and saving in the view of their expected financial situation. Firms’ sentiment is instead viewed from a business perspective, looking at the future developments of production, order books and stocks. The problem of measuring and monitoring this sentiment about the current and future state of the economy became a topic of absolute relevance to any policymaker for designing timely policy responses, especially in the presence of shocks (e.g., the COVID-19 pandemic).

In recent years, in addition to classic macroeconomic *leading* indicators (for a comprehensive list of indicators see Altissimo et al. 2010; Frale et al. 2011), new promising indicators have been proposed to monitor and short-term forecast economic activity. In particular, a great debate in the literature concerned the role of confidence indicators^1^ at this aim (e.g., Mourougane and Roma 2003; Banerjee et al. 2005; Taylor and McNabb 2007). Nevertheless, measuring public sentiment is a hard task, with diverse approaches discussed in the literature. Becker et al. (2017), Ardia et al. (2019) and Larsen and Thorsrud (2019) proposed, for example, to measure sentiment on the basis of newspaper articles. However, if newspapers surely express the media sentiment about the economy, they do not necessarily reflect the sentiment of people and firms. A recent proposal considered alternatively a sentiment measured through social media (e.g., Li et al. 2017). However, this approach has to deal with some critical issues such as spamming, the presence of automatic bots, fake accounts and fake news (see Ferrara et al. 2016; Antonakaki et al. 2021). More traditionally, in a macroeconomics framework, the analysis of confidence surveys and the resulting *confidence indicators* (e.g., Banerjee et al. 2005; Taylor and McNabb 2007; Gelper and Croux 2010; Sorić et al. 2016) are considered. A different choice is to investigate people’s and firms’ opinions directly, with certified business and consumer surveys, interviewing them about their current economic situation and future expectations.

The latter approach offers the possibility of constructing a statistical measure of the sentiment, known as *confidence indicator*. For the different involved entities, the confidence indicators provide a picture of the sentiment towards the economy. The different indicators—at a consumer level and at a firm-level—can be subsequently combined to obtain an overall measure using a composite indicator. To the best of our knowledge, the EU is the only institution that aggregates the confidence indicators related to the different sectors in a composite confidence indicator known as *Economic Sentiment indicator* (ESI), to monitor public sentiment about the economy. The ESI is produced by the European Commission’s Directorate General for Economic and Financial Affairs (DG ECFIN), following the *Joint Harmonised EU Programme of Business and Consumer Surveys* (European Commission 2020). The adopted weighting scheme is defined by following a *relevance criterion*, considering the relative contribution of the consumers and the different business sectors to the Gross Domestic Product (GDP). The GDP represents a fundamental measure of the health state of an economy. However, while policymakers require timely information about the economic activity, the GDP is observed quarterly and published with a considerable lag, usually many months. For this reason, many institutes (e.g., central banks, statistical institutes) started to collect other high-frequency variables, aiming at monitoring the state of the economy in a shorter time (Aruoba et al. 2009).

Nevertheless, there are several drawbacks to the weighting scheme of the ESI. First of all, the weights are not country-specific because they are the same for each economy surveyed in the EU. In other words, the relevance criterion is computed by looking at European aggregates instead of national ones. Second of all, the relevance criterion does not allow using dynamic weights. Confidence indicators are weighted almost in the same way because the contribution of a sector may change in the long run by considering the productive structure evolution of the analysed economy. The ability of sector sentiments to generate fluctuations in the GDP, highlighted by several studies (Zanin 2010; Qiao et al. 2009; Gelper et al. 2007), is indeed not taken into account by the relevance criterion. Conversely, since each sector has a specific business cycle, confidence indicators could be differently weighted over time, especially for business sectors highly dependent on fluctuations (e.g., the construction). It could be, for example, that shocks in the sentiment level of a sector less contributing to GDP have a more significant impact than in the sentiment level of a sector with a higher contribution. Similarly, shocks in the sentiment level of consumers could become more critical during uncertainty periods with respect to other periods of relative economic stability. Finally, another drawback relies on the information encompassed by ESI. The ESI does not consider the media sentiment even if it is a crucial aspect to consider. For example, as pointed out by Blood and Phillips (1995), during the 1992 US presidential election, the media were accused by some economists to have negatively highlighted the political and economic situation, causing negative economic consequences for the country. According to the author, the negative economic coverage played a determinant role in the delay of the anticipated economic upturn, a phenomenon defined as “media malady”. All these limits could make the ESI not the best solution for correctly monitoring the public sentiment in a short time, and less accurate if taken as a leading indicator in forecasting (see Gelper and Croux 2010; Sorić et al. 2016).

This paper proposes a new approach for constructing a composite confidence indicator. We developed, in particular, two different alternative solutions. The first one, called Mixed Frequency Confidence Composite Indicator (MF-CCI), relies on the same information set of ESI, but every single component is dynamically weighted according to its impact on GDP over time, using a direct approach. Since the GDP is quarterly observed while the confidence indicators are monthly sampled, we use a *mixed-frequency regression model* to handle mixed-frequency data (Foroni and Marcellino 2013). The second one, called *Mixed Frequency Confidence Composite Indicator Plus* ($\text{MF-CCI}^{+}$), is built by also introducing media sentiment together with business and consumer confidence. Thus, we consider a composite confidence indicator based on an expanded information set in the latter case. The proposed indicators have several applications. They can be used to monitor sentiment reaction to exogenous shocks and policy responses, allowing policymakers to evaluate the impact of policies on public opinion. At the same time, they can be used as short-term indicators to forecast economic activity. The two proposed indicators have been tested for some Euro Area economies, including Belgium, France, Germany, Greece, Italy and Spain. We used the MF-CCI and the $\text{MF-CCI}^{+}$ for evaluating the sentiment evolution during the COVID-19 shock. In particular, we analysed the public’s reaction after the restrictions introduced in the different waves of the pandemic for reducing the spread of COVID-19 contagion. With this respect, we find that the sentiment dramatically reduced around almost all countries after introducing strict policies like the lock-downs. However, public opinion’s reaction was much more pessimistic during the first wave than in the second. The effectiveness of the proposed indicators has been further discussed by considering an empirical study on GDP forecasting. To this aim, we considered the mixed-frequency-based approach of Ghysels et al. (2016) because of the presence of mixed-frequency data. The results showed an increasing forecasting accuracy when MF-CCI and $\text{MF-CCI}^{+}$ are employed.

The paper is structured as follows. Firstly, we provided in Sect. 2 a brief review of the methods proposed in the literature for measuring the public economic sentiment. Then, the novel proposed approach is discussed in detail in Sect. 3. Section 4 presents a case study applying the novel public sentiment indicators by exploring people’s and firms’ reactions to public policies after the COVID-19 shock. Section 5 provides a discussion about the usefulness of this new class of sentiment indicators, also showing how these indicators can be used to forecast GDP in the selected countries. Finally, some remarks about the proposal and future developments are presented.

## Measuring the public sentiment

Several ways of measuring the sentiment about the economy are currently available. Most of the newest approaches take advantage of machine learning techniques, especially those of *text mining*. For example, Becker et al. (2017) were the first to develop a measure of media’s perception about economic uncertainty. They collected article published on the major US newspapers containing terms like “uncertainty”, “economy”, “Congress”, “deficit”, “Federal Reserve”, “legislation”, “regulation” and “White House” (including variants like “uncertainties”, “regulatory” or “the Fed”). The authors searched on the digital archives of each journal to obtain a monthly count of articles. Since the overall volume of documents varied across newspapers and time, they standardised each monthly newspaper-level series to unit standard deviation and then computed the monthly averages. In this way, they developed an indicator called the *Economic Policy Uncertainty* (EPU), which is a measure of media sentiment linked to the concept of uncertainty. Ardia et al. (2019) applied text mining techniques to conduct sentiment analysis on the US newspaper textual content. In particular, the authors computed a textual sentiment indicator by retrieving the set of articles from the US newspapers under six clusters of topics that identify economic concepts (“GDP output”, “Job market”, “Prices”, “Interest rate”, “Real estate”, “Surveys”). The authors showed that using this sentiment indicator improves the accuracy of the US industrial production forecasts. More recently, Larsen and Thorsrud (2019) developed a business cycle indicator considering Norwegian newspaper articles and applying text mining techniques. Even if these approaches are very promising, we note that the newspaper articles may strictly represent only the media sentiment and do not evaluate the overall public sentiment. Another interesting use of text mining techniques relies on the sentiment computed from social media, where people can directly express their opinions in a written form. This second approach has been successfully applied, for example, to predict the stock price movements (Li et al. 2017). Nevertheless, also in this case, there are critical drawbacks because of the so-called *social hacking* problem, i.e. manipulating opinion dynamics by disseminating fake contents (Stella et al. 2018). The *social hacking* relies on practices such as spamming, automatic bots, fake accounts, fake news, hate speeches and so forth (see Ferrara et al. 2016; Fortuna and Nunes 2018; Antonakaki et al. 2021). In other words, the information coming from social media is not trustworthy if not properly filtered.

More traditionally, policymakers use the results of confidence surveys to measure public opinion orientation towards the state of the economy. The availability of trustworthy information is crucial for policymakers, and this is one of the main reasons public sentiment is still measured employing official surveys certified by National Statistical Institutes (Banerjee et al. 2005; Taylor and McNabb 2007; Gelper and Croux 2010; Sorić et al. 2016). These surveys provide in-depth information on various aspects of business economic activity and consumer behaviour. Since the beginning of the 1980s, confidence surveys have been regularly carried out monthly and quarterly in several countries. Concerning EU, distinct harmonised surveys are intended for the manufacturing industry, construction, retail trade, services, financial services (investments), as well as consumers, even if some countries do not carry on all the surveys or merge some of them. The sample size for each national survey varies and is generally related to the corresponding population size. At present, about 23,000 consumers and 95,000 firms (26,000 units for industry, 31,000 units for services, 21,000 units for retail trade and 17,000 units for construction) are surveyed monthly across the EU, with a non-response rate of around 30%. Answers obtained from the surveys are then aggregated in the form of *balances*, differences in the percentages of respondents giving positive and negative replies, on which a seasonal adjustment is also performed (Driver and Urga 2004; Claveria et al. 2017). Balances allow the presentation of a single value as a summary of the different responses and the representation of changes in those responses over time through a single time series. A confidence indicator is calculated as the arithmetic mean of the seasonally adjusted balances for each sector.

As stated in the introduction, the EU is the only institution that computes a composite confidence indicator based on the business and consumer surveys information. The weighting scheme adopted by the ESI considers a *relevance criterion* (Gelper and Croux 2010), considering the representativeness of each sector in terms of its contribution to the aggregated GDP. Following this logic, a 0.40 weight is assigned to the industrial confidence indicator, a 0.30 weight to the services confidence indicator, a 0.05 weight to both the retail trade confidence indicator and the construction confidence indicator, and a 0.20 weight to the consumer confidence indicator. The weights mentioned above are not directly applied to the five confidence indicators themselves but to their standardised individual component series. A summary of the ESI components is reported in Table 1. Weights have been arbitrarily chosen by the European Commission and have not experienced any major revision in the last years. Moreover, all the EU countries adopt the same weights such that the weighting scheme proposed by the European Commission is not country-specific. Several authors discussed the forecasting power of ESI (Gausden and Hasan 2020) and analysed the evolution of the ESI at a country or EU level (Zanin 2010; Ghonghadze and Lux 2012), without questioning about its construction.

**Table 1:** Structure, weight and time frequency of the ESI components

Component	Weight	Indicators	Time references
Industrial confidence	0.40	Overall order books	Current
		Stocks of finished products	Current
		Production to develop	Over next 3 months
Services confidence	0.30	Business situation develop	Over past 3 months
		Changes in demand of services	Over past 3 months
		Expectations in demand of services	Over next 3 months
Retail trade confidence	0.05	Sales develop	Over past 3 months
		Volume of stock	Current
		Expectations in sale changes	Over next 3 months
Construction confidence	0.05	Order books	Current
		Expectations in employment changes	Over next 3 months
Consumer confidence	0.20	Changes in financial situation	Over past 12 months
		Changes in financial situation	Over next 12 months
		General economic situation	Over next 3 months
		Expectations in expenditure	Past/next 12 months

Few authors analysed the shortcomings of the ESI weighting scheme, proposing alternative solutions. Gelper and Croux (2010) analysed the ESI weighting scheme and proposed new kinds of aggregation, comparing the performances of an ESI obtained with a *Dynamic Factor* (DA) approach developed by Stock and Watson (2002) and a *Partial Least Squares* (PLS) approach (see Esposito Vinzi et al. 2010). The authors proved that the PLS estimator outperforms both the official ESI and the DF estimator by analysing the industrial production series. Noteworthy, results are not robust in terms of forecasting accuracy, and the proposed approaches did not show a significant improvement with respect to official ESI. Moreover, the selection of weights is conducted regardless of the business cycle fluctuations and the relationship between confidence and GDP, leading to weights that may be potentially meaningless from an economic standpoint. Sorić et al. (2016) proposed a weighting scheme for the ESI components based on *nonlinear optimisation with constraints*, deviating from the classical linear aggregation approach widely used in composite indicator building (Freudenberg 2003). A remarkable innovation of the latter proposal relies on the expedient used to deal with time-frequency since, usually, GDP data are available every quarter. In particular, the authors carried on the Chow and Lin (1971) temporal disaggregation method to estimate GDP every month. This approach has some critical flaws as well. First of all, the choice of regressors used to disaggregate GDP temporally is arbitrary, and the disaggregation has to be improved by considering additional or alternative covariates in the model. Moreover, the procedure is based on an *ex-ante* evaluation of prediction errors. In a similar framework, focusing on professionals’ and experts’ views on economic developments obtained through the *World Economic Survey* (Boumans and Garnitz 2017), Claveria et al. (2018) proposed a temporal aggregation approach to forecast GDP. In particular, they used *symbolic regression*, an empirical modelling approach not relying on a specific prior structure.

In the presence of a frequency mismatch, it is well known that mixed-frequency regression models are better suited (Foroni and Marcellino 2013). Since we deal with the quarterly GDP and a set of monthly confidence indicators, it is desirable to handle mixed-frequencies without recurring to temporal aggregation or disaggregation, avoiding the loss of information caused by the first approach and the intrinsic uncertainty caused by the second one. For this reason, we propose building a composite confidence indicator by considering a weighting scheme derived from a mixed-frequency model. Each component is dynamically weighted according to its impact on GDP over time.

## Mixed-frequency composite confidence indicators

Composite indicators are used in several domains to summarise the information coming from different sources in a meaningful way. These indicators are obtained by synthesising single sub-indicators under a model that underlies the multi-dimensional concept to be measured (e.g., Freudenberg 2003; Mazziotta and Pareto 2016; Decancq and Lugo 2013). The construction of a composite indicator is based on several steps (Greco et al. 2019): (i) the choice of a theoretical framework referring to the concept we want to measure; (ii) the identification of sub-indicators and groups of sub-indicators (also known as *components)* expressing the different dimensions to take into account; (iii) the selection of an aggregation method.

In the case of composite confidence indicators, we consider public opinion about the economy as a theoretical framework. The selection of the individual sub-indicators has been provided in Sect. 2, discussing the different approaches for measuring the sentiment. In particular, following the macroeconomic tradition, we consider business and consumer confidence surveys as trustworthy information about firms and people’s beliefs about the current and future state of the economy. Moreover, we consider a measure of the media sentiment as an additional sub-indicator, choosing the EPU. This indicator is easy to construct and has been proved to help analyse the media sentiment about the economy (see Perić and Sorić 2018; Tobback et al. 2018; Ghirelli et al. 2019). Even if the EPU is commonly calculated for many countries worldwide, a limit could be its availability for a fraction of the EU economies, constraining the studies focused on the whole EU.

The different sub-indicators used to construct the composite indicator can have different measurement scales and polarities. The polarity depends on the sign of relation between a single indicator and the phenomenon to be measured (i.e., the sentiment). We consider a min-max standardisation to overcome this problem, assigning a positive polarity to the surveys-based confidence indicators and a negative one to the EPU. Indeed, an increment in the survey-based confidence indicators is related to an increase in the sentiment levels, whereas an increase in the EPU means that the media sentiment decreases because of increasing uncertainty.

The subsequent step relates to the aggregation method. As in most of the previous literature, we employ an objective perspective rather than a subjective one. In a subjective approach, weights are defined following an expert knowledge about the investigated phenomenon. An obvious limit of subjective approaches is the arbitrariness of experts in the weight assignment process, leading in extreme cases to an outcome manipulation (Grupp and Mogee 2004). Following an objective approach, instead, the weights employed for sub-indicators or components are obtained through a data-driven approach (Decancq and Lugo 2013). When regression analysis is chosen to deal with this task, weights can be derived by analysing for each sample unit *t* $(t = 1, \dots , T)$ the relation between an endogenous criterion $y_t$ and a set of predictor observations $x_{it}$ $(i = 1, \dots , N)$:

1 $$\begin{aligned} y_t = f(x_{it};\;\beta _{it}) + \epsilon _t \end{aligned}$$

where $f(\cdot )$ is a function modelling the assumed relationship, $\beta _{i,t}$ the regression coefficients and $\epsilon _t$ is the error term representing the non-modelled determinants of $y_t$. Given a linear model in which the endogenous variable is represented by the GDP, Eq. 1 can be written as:

2 $$\begin{aligned} GDP_t = \beta _{1t}x_{1t} + \dots + \beta _{Nt}x_{Nt} + \epsilon _t \end{aligned}$$

The $\beta _{it}$ in Eq. 2 are commonly estimated via ordinary least squares (OLS):

3 $$\begin{aligned} \min _{\pmb {\beta }} \sum _{t=1}^{T} (GDP_t - \beta _{1t}x_{1t} + \dots + \beta _{Nt}x_{Nt})^2 \end{aligned}$$

The scaled estimated coefficient ${\hat{w}}_i = {\hat{\beta }}_i/\sum _{i=1}^{N}{\hat{\beta }}_i$ can be interpreted as the weight associated to the $x_i$ component. An alternative approach is to minimise Eq. 2 under the constraint $\sum _{i=1}^{N} \beta _{i} = 1$. However, when the $x_{it}$ indicators are recorded with a different frequency with respect to $y_t$, it is not possible to apply the method previously described. As an example, the ESI is observed on a monthly basis, while GDP is usually observed every quarter. To overcome the problem caused by the different time-frequency, it is possible to use a temporal aggregation by considering the monthly observations quarterly or a temporal disaggregation by estimating the GDP monthly. As stated above, temporal aggregations/disaggregations induce a consistent loss of information and biased results (Marcellino 1999). Other approaches can be considered to estimate statistical relations under a frequency mismatch. The main proposals dealing with this issue are based on *bridge models* (Baffigi et al. 2004) and *mixed-data sampling* (MIDAS) regression (Ghysels et al. 2007). Bridge models are specifically applied in forecasting problems where a frequency mismatch has to be considered, predicting a low-frequency endogenous variable by low-frequency lags of a set of predictors. The obtained forecasts are aggregated and used as covariates in a regression model with only low-frequency data. In MIDAS regressions, on the other hand, the observations of the low-frequency variable are directly related to lagged high-frequency predictors without considering time aggregations. Marcellino and Schumacher (2010) showed that MIDAS models perform better than bridge models in forecasting problems. MIDAS is a direct multi-step forecasting tool, whereas bridge equations are mostly based on iterated multi-step forecasts from an additional high-frequency model. Moreover, MIDAS employs empirical weighting of high-frequency predictor observations often based on functional lag polynomials, whereas bridge equations are based on fixed weights derived under statistical time aggregation rules (Kvedaras and Račkauskas 2010; Foroni and Marcellino 2013).

Taking into account the mixed-frequency theoretical framework, it is possible to build an ESI-like confidence composite indicator based on monthly observations. Differently from the original ESI and its alternatives based on time aggregations or disaggregations previously recalled, the weighting scheme here introduced is derived from a MIDAS regression model.

### The mixed-frequency regression model

The MIDAS models allow predicting a low-frequency dependent variable *y* of length *T* by a number of higher frequency variables $x_{i}$ of length *mT*—where *m* is the observed frequency mismatch—through an operation called *frequency alignment* (see Ghysels et al. 2016). The frequency alignment is used to transform each high-frequency vector $\mathbf{x_{i}}$ with *mT* elements into a low-frequency matrix $\mathbf{X_{i}}$ with *T* rows and *m* columns known as *stacked vectors*. Thus, the mapping of MIDAS follows a simple time-ordering aggregation scheme. Since GDP is observed on a quarterly basis, it is possible to transform a high-frequency variable $\mathbf{x_i}$ (e.g., a confidence indicator recorded on a monthly basis) in a low-frequency matrix $\mathbf{X_i}$ with 3 stacked vectors:

4 $$\begin{aligned} \mathbf{x_{it}} = \left[ \begin{array}{c} x_{i1} \\ x_{i2} \\ \vdots \\ x_{i(3T)} \end{array}\right] \rightarrow \left[ \begin{array}{ccc} x_{i3} &{} x_{i2} &{} x_{i1}\\ x_{i6} &{} x_{i5} &{} x_{i4}\\ \vdots &{} \vdots &{} \vdots \\ x_{i(3T)} &{} x_{i(3T-1)} &{} x_{i(3T-2)} \end{array}\right] = \mathbf{X_{it}} \end{aligned}$$

By applying the frequency alignment to the monthly-recorded single indicators, the model specification of the proposed strategy can be written as:

5 $$\begin{aligned} GDP_t = \sum _{j=1}^{3} \sum _{i=1}^{I} \beta _{ij} x_{i(3t-j)} + \epsilon _t \end{aligned}$$

where $y_t$ is the GDP time series and $x_{i(3t-j)}$ are the stacked vectors.

When the frequency mismatch *m* is not so huge (e.g., $m = 3$), as in our case with a GDP observed each quarter and a composite indicator recorded each month, Foroni et al. (2015) and Ghysels and Qian (2019) showed that the parameters in Eq. 3 could be easily estimated via OLS. The weights can be computed by normalising the estimated coefficients. However, in the light of the work of Sorić et al. (2016), weights can be derived as the solution to a constrained optimisation problem. In particular, the weights vector $\beta$ is obtained by minimising the following objective function:

6 $$\begin{aligned}&\min _{\beta }: \left( y_t - \beta {\mathbf{X}}_{it}\right) ^2 \\&\text{s.t.} \sum _{j=1}^{3} \sum _{i=1}^{I} \beta _{ij} = 1, \beta _{ij} \ge 0 \forall i,j \nonumber \end{aligned}$$

where ${\mathbf{X}}_{i,t}$ is the matrix containing the sub-indicators stacked vectors from (4), $\beta$ is the vector containing the $3 \times I$ weights and each $\beta _{ij}$ represents the weight that has to be assigned to each *i*-th component within every high-frequency period. Indeed, once parameters are estimated, an additional step is required in the case of mixed-frequency data. The weight associated with the *i*-th component (namely, ${\hat{w}}_i$) is equal to the sum of the weights computed within the low-frequency period:

7 $$\begin{aligned} {\hat{w}}_i = \sum _{j=1}^{3} {\hat{\beta }}_{ij} \end{aligned}$$

It is essential to underline that all the ${\hat{\beta }}_{ij}$ are used, instead of averaging them, to ensure that the weighted sum is equal to unity. It is noteworthy that the optimisation problem (6) can be seen as a constrained least square (CLS) problem with mixed-frequency data.

The *Mixed-Frequency Composite Confidence Indicator* (MF-CCI) is obtained by weighting each component $x_{it}$:

8 $$\begin{aligned} \text{MF-CCI}_t = \sum _{i=1}^{I} {\hat{w}}_i x_{it} \end{aligned}$$

As in the ESI scheme, the MF-CCI considers only the survey-based confidence indicators ($I=5$). Including the EPU as an additional media sentiment indicator ($I=6$), it is possible to obtain another composite indicator identified in the following as $\text{MF-CCI}^{+}$.

## A case study: the sentiment in EU during COVID-19 pandemic

As stated in the introduction, a composite confidence indicator can be helpful to monitor the public opinion orientation towards the economy. A good indicator has to provide a reliable picture of economic agents’ sentiment, especially during periods of crisis. The shock induced by COVID-19 represents an interesting example of this need. Since the first case in China, the COVID-19 rapidly spread worldwide, becoming a pandemic with millions of infections and deaths. The spread of the disease among the EU countries did not follow a uniform spatio-temporal pattern (Ehlert 2021), but almost all the countries replied with severe restrictions (e.g., lock-downs) to contain the contagion effects. The governments’ efforts were massive. For example, in Italy, a total of 761 legislative acts were issued to contrast the pandemic between January 2020 and September 2021, with an average of 37 acts per month.^2^

The introduced policies had a powerful negative psychological impact on citizens (Yao et al. 2021), clearly affecting consumer behaviours (Amicarelli et al. 2021; Bender et al. 2021) and preferences (Filimonau et al. 2021; Vargas-Lopez et al. 2021). Therefore, measuring public opinion sentiment became a crucial issue (Alaimo et al. 2021). Media sentiment towards economic policy reduced, and subsequently, the uncertainty increased as well (Caggiano et al. 2020). Nevertheless, it is not clear how the restrictions employed by the EU governments affected the public opinion and if this impact was homogeneous among the different waves of contagion. Therefore, we decide to compare the evolution of the economic sentiment during the COVID-19 pandemic either employing the ESI, the MF-CCI and its extension for media sentiment $\text{MF-CCI}^{+}$. Since the ESI weights are constant, we expect that it leads to imprecise evidence about the evolution of the sentiment over time in the presence of huge shocks.

Here we considered a sample including both three “virtuous” economies—Belgium, France and Germany—and three so-called PIIGS economies^3^—Greece, Italy and Spain—. We collected, for each selected country, GDP and confidence data as well as the EPU data,^4^ considering as time interval the period between the $1^{st}$ of January 2001 and the $1^{st}$ of March 2021, because of the data availability for all the countries and the necessity of including the years before and after COVID-19 spread. All the computations have been implemented with the version 4.0.2 of the R software.^5^ The GDP is accounted as growth rate, on the basis of country-specific values expressed in current prices, while the confidence indicators are considered at their levels.

**Table 2:** Main descriptive statistics for each analysed country

	Mean	St.Dev.	Min	Max		Mean	St.Dev.	Min	Max
*Belgium*					*Greece*				
GDP growth (%)	0.33	± 1.98	− 11.93	11.77	GDP growth (%)	− 0.08	± 2.27	− 12.89	4.42
Consumer conf.	− 6.80	± 4.68	− 20.10	5.40	Consumer conf.	− 7.39	± 7.09	− 24.90	3.60
Industrial conf.	− 7.56	± 7.92	− 33.80	6.90	Industrial conf.	− 4.59	± 11.99	− 42.20	16.40
Services conf.	8.73	± 14.85	− 50.10	32.20	Services conf.	12.13	± 13.22	− 30.80	45.70
Retail conf.	− 5.96	± 8.81	− 43.00	13.70	Retail conf.	− 14.17	± 11.28	− 41.60	13.50
Construction conf.	− 8.35	± 8.21	− 24.70	7.40	Construction conf.	− 18.13	± 21.39	− 55.70	22.60
EPU	104.88	± 40.94	58.95	368.81	EPU	150.32	± 79.07	28.43	498.06
*France*					*Spain*				
GDP growth (%)	0.26	± 2.67	− 13.21	18.53	GDP growth (%)	0.29	± 2.90	− 17.78	17.05
Consumer conf.	− 14.46	± 6.23	− 31.60	− 1.00	Consumer conf.	− 39.31	± 21.07	− 80.80	3.20
Industrial conf.	− 4.30	± 8.36	− 34.40	12.80	Industrial conf.	− 6.26	± 10.87	− 36.60	13.30
Services conf.	1.52	± 13.27	− 52.50	33.10	Services conf.	4.12	± 24.14	− 46.80	58.70
Retail conf.	− 3.09	± 11.20	− 40.50	17.40	Retail conf.	− 0.27	± 20.69	− 48.40	39.00
Construction conf.	− 17.92	± 11.18	− 41.00	4.60	Construction conf.	− 29.60	± 26.63	− 77.40	32.50
EPU	114.79	± 37.16	54.16	261.61	EPU	99.53	± 27.65	37.70	188.70
*Germany*					*Italy*				
GDP growth (%)	0.24	± 1.72	− 9.69	8.70	GDP growth (%)	− 0.028	± 2.46	− 12.93	15.86
Consumer conf.	− 10.56	± 5.32	− 22.30	2.40	Consumer conf.	− 14.12	± 10.58	− 41.70	2.50
Industrial conf.	− 6.27	± 9.35	− 39.30	14.70	Industrial conf.	− 7.27	± 8.97	− 39.70	5.50
Services conf.	− 0.53	± 10.44	− 45.60	14.60	Services conf.	5.39	± 22.14	− 53.30	47.80
Retail conf.	− 7.39	± 6.97	− 37.30	8.80	Retail conf.	− 5.82	± 13.48	− 38.50	17.00
Construction conf.	− 5.65	± 22.82	− 47.50	44.90	Construction conf.	− 14.73	± 26.73	− 69.30	39.00
EPU	193.97	± 99.59	30.62	574.63	EPU	114.91	± 41.74	31.70	279.39

GDP is expressed in percentage points, the other variables are expressed in their levels

**Fig. 1:**
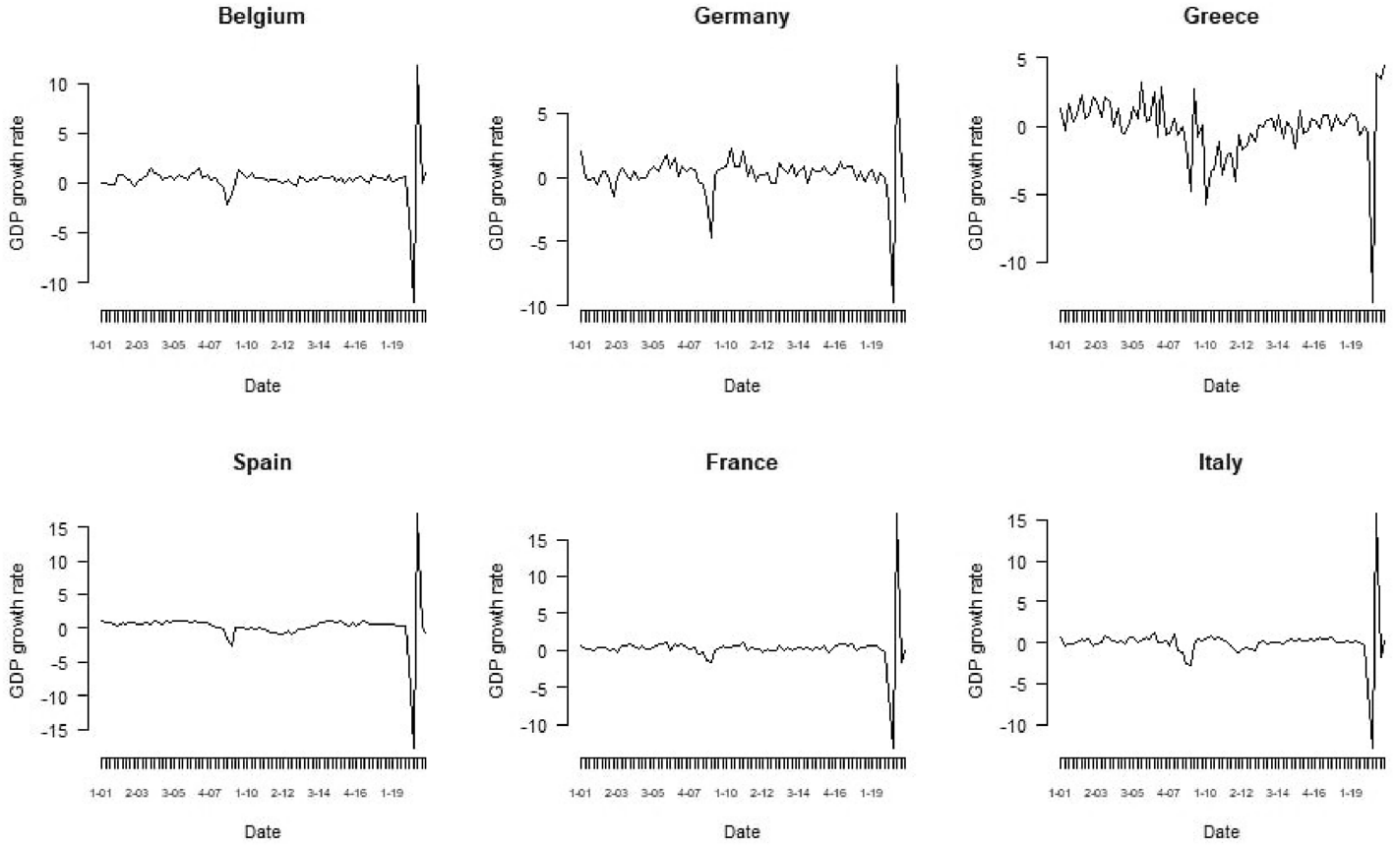
GDP growth rates for the considered countries

Table 2 reports the descriptive statistics of the considered dimensions. All the countries experimented minimum negative GDP growth rates, corresponding to the first quarter after the beginning of the pandemic, recovering in the subsequent quarters. The time evolution of the GDP growth rates in Fig. 1 highlighted a greater impact of the COVID-19 shock than the one caused by the global financial crisis, as also stated by Ambrocio (2021) for consumer and business confidence separately.

The confidence indicators showed peculiar country-specific characteristics. The only exception is represented by the services sector, the only one with a positive average and the highest variability across the different business sectors for all the countries. Countries like France and Belgium showed similar variability in the media sentiment (i.e., the EPU), while others like Germany and Greece were more volatile. Similarly, countries like Spain and Italy had a low average value of consumer confidence, while the consumers of other economies—such as Belgium—were much more confident on average. Moreover, virtuous economies showed very low variability in consumer confidence. This evidence suggests that even in the presence of massive shocks, the consumer confidence of these countries is less affected than the others. We conclude that changes in the GDP must be considered in the construction of an aggregate sentiment indicator where, instead, the ESI adopts a static perspective, not considering any fluctuation in the computation of sub-indicators weights.

**Fig. 2:**
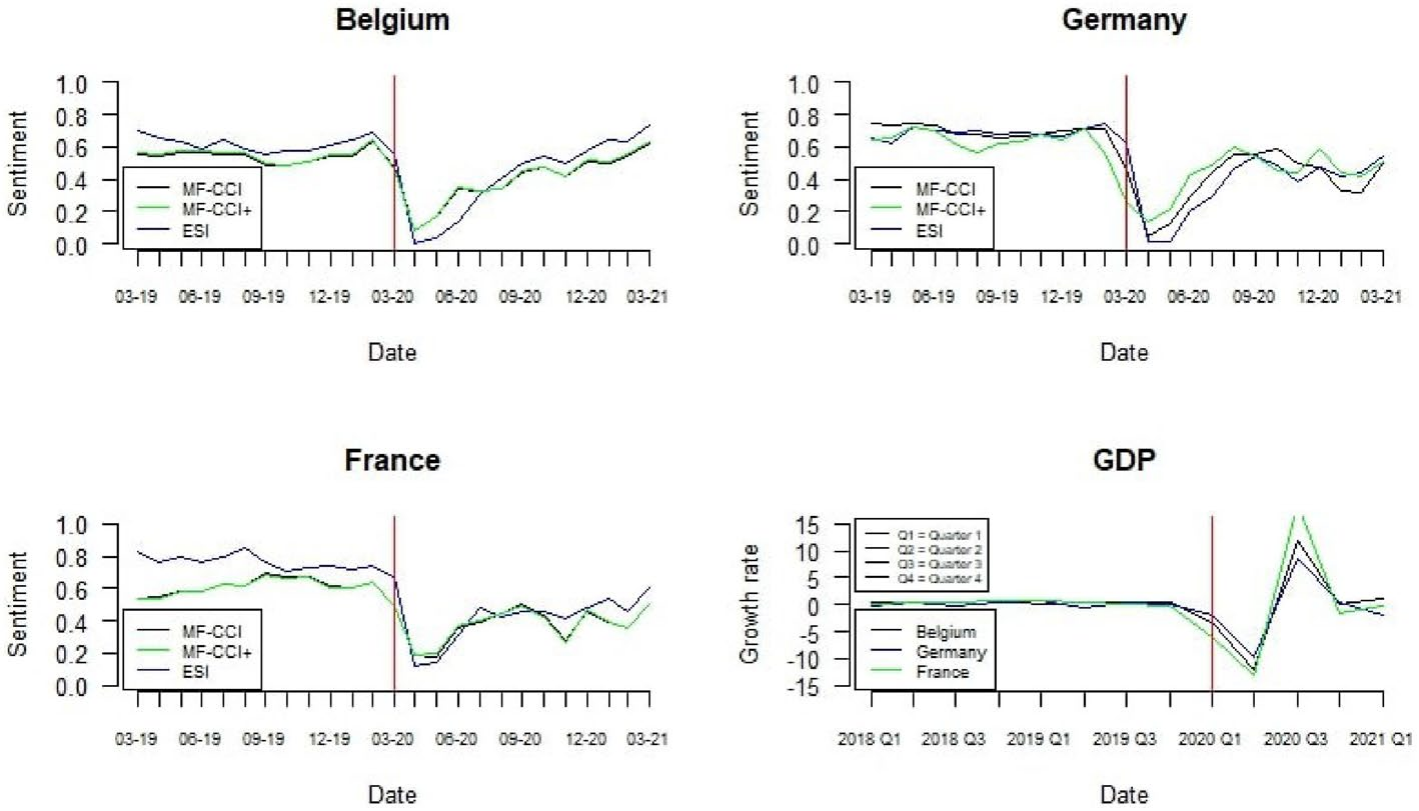
MF-CCI (black line), $\text{MF-CCI}^{+}$ (green line) and ESI (blue line) evolution for Belgium, France, Germany (Vertical red line indicates March 2020)

In order to better understand the dynamic of the public sentiment during the pandemic, we mainly focused on the period between the $1^{st}$ March 2018 (i.e., two years before the pandemic) and $1^{st}$ March 2021. Fig. 2 reports the time patterns of the ESI, the MF-CCI and the $\text{MF-CCI}^{+}$ for Belgium, France and Germany. All the indicators are normalised. Furthermore, the GDP growth rates evolution for the three countries within the same period is reported.

We observed that starting from March 2020, the confidence indicators suddenly decreased and, simultaneously, the GDP growth rate started to reduce. The minimum value for all the variables was reached during the second quarter of 2020. Belgium registered the first confirmed COVID-19 case on the $4^{th}$ of February 2020. Like most EU countries, the government developed different measures to limit the contagion, such as closing schools and non-essential businesses and limiting not essentials movements. Accordingly, a decrease in the sentiment has been registered as shown in Fig. 2. Then, in May, the government announced diminishing restrictions. After such an announcement, sentiment increased rapidly. However, because of a subsequent huge increase in cases, new restrictions were imposed in October 2020, with a new lock-down from November 2020. We note that the MF-CCIs captured small rises and falls in the sentiment series (i.e., a fall after introducing the second lock-down and a rise after its removal), while the ESI showed an ever-increasing curve after April 2020.

France experimented the first lock-down in the middle of March 2020, with the first COVID-19 case registered at the end of January. After this date, as in the other countries, we observed a considerable reduction in the sentiment. The government relaxed the first round of containment measures in May 2020, but based on regional differences. Therefore, sentiment started to rise, with the maximum point reached in June 2020, where most restrictions stopped. The rise of infections in August 2020 led to new restrictions associated with a novel reduction in the sentiment, even if lower than in the first wave. Then, sentiment became stable, and all the indicators well capture this aspect. However, ESI did not capture the reduction in sentiment due to the restrictions introduced to contain the contagion during the second wave. On the contrary, the MF-CCIs indicators showed a decline in the sentiment due to the second lock-down.

Germany registered the first COVID-19 case in January 2020. Then, the country experimented some restrictions, with school and business activities closures, social distancing and a ban on public gatherings. After March 2020, all the indicators reported a considerable decrease in the sentiment values. In the light of the increasing pandemic spread, the government introduced a strict lock-down from the beginning of 2021 until March 2021. While ESI showed an increase in the sentiment from November 2020 to March 2021, the MF-CCI reported a constant decrease in the values. On the other side, the $\text{MF-CCI}^{+}$ highlighted the importance of media in Germany. The German GDP fell less than in other countries during the pandemic, and the increase in media sentiment in the last months of 2020 was likely due to this evidence.

**Fig. 3:**
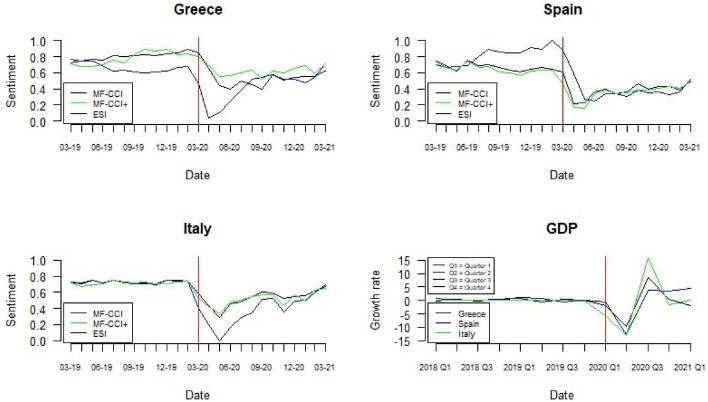
MF-CCI (black line), $\text{MF-CCI}^{+}$ (green line) and ESI (blue line) evolution for Greece, Italy, Spain (Vertical red line indicates March 2020)

In Fig. 3, we considered the time patterns of the three indicators for the other three analysed countries, Greece, Italy and Spain.

The first confirmed COVID-19 case in Greece was reported in February 2020. Since then, the government has adopted strict containment measures during the second quarter of 2020. All the indicators recognised a decrease in the sentiment after introducing these measures. However, the change in the sentiment was more severe for the ESI than the developed MF-CCIs. The ESI recorded an impressive increase in the sentiment during the lock-down, while the MF-CCI suggested that the public sentiment remained stationary around the minimum point. Then, the government announced a new lock-down starting after summer 2020. While the ESI showed a positive trend, the MF-CCIs indicators registered an additional decrease in the sentiment.

Italy was one of the most affected countries during the COVID-19 pandemic. Different measures have been introduced at a national and a regional level to prevent and contain the contagion (Panarello and Tassinari 2021). In March 2020, Italy experimented a first lock-down. Accordingly, the sentiment reduced immediately. Then, the lock-down expired at the beginning of May 2020 and the sentiment raised. Nevertheless, at the beginning of November 2020, the government introduced new measures based on a differentiated regime of restrictions among the Italian regions. Furthermore, the reduction of the sentiment in the second wave of contagion was much lower than in the first one. The $\text{MF-CCI}^{+}$ better showed this reduction in the sentiment. This evidence suggests the importance of considering media as an additional information source.

Spain experienced the first COVID-19 case at the end of February 2020. From March 2020, the government introduced severe restrictions, and all the indicators highlight a consequent decrease in the sentiment. Nevertheless, Spain seemed to be the only country where the sentiment was not much negatively affected during the second wave of contagion. Indeed, after the renewed restriction was introduced to limit the pandemic spread, we observed a timid decrease and subsequently an increase in the sentiment. Subsequently, it became almost stable over time. In general, the reduction of sentiment during the first wave was much more substantial than in the second one.

## Discussion

One of the most important advantages of confidence measures—as stated above—is the speed at which data are available. In most countries, the processing time for obtaining a measure of the sentiment at a consumer or a firm-level is less than a month, where official statistics are instead released with delays of two to three months or more. Another critical aspect, apart from the timeliness, is that sentiment data are less volatile because they are based on qualitative judgements—in the form of opinion polls—hence they are free of the distortions that may affect official statistics (Santero and Westerlund 1996), like climate conditions. On the other hand, sentiment measure encompasses subjective information that can be interpreted in different ways, so that the relationship between economic variables and confidence indicators has to be carefully evaluated. However, the early signal of tendencies or turning point into the reference economy offered by confidence indicators can help policymakers orient their decisions, waiting for quantitative data. This earliness is particularly vital when fast and unexpected shocks occur. As showed, before March 2020, the ESI and the MF-CCI almost overlapped and had the same trend for all the analysed countries, with few exceptions. After March 2020, we observed that the time pattern of ESI was often very different from the MF-CCIs indicators’ ones since the ESI did not take into account the impact of each sector’s confidence on the GDP growth during the contagion waves.

A policy report of the European Commission published on March 2021,^6^ entitled *Impacts of the COVID-19 pandemic on EU industries*, highlighted that the EU industries have been differently affected by the pandemic. A clear example is the construction sector. For example, in Germany, it was possible to continue construction activity more or less as before, while in some countries (e.g., Italy, Spain and France), the construction activity was severely limited. Hence, static weights for each country are not appropriate, especially with sectors highly dependent on the business cycle. The MF-CCIs indicators cope with this limit providing country-specific weights based on the impact of people’s and firms’ sentiment over the GDP.

Evaluating the forecasting accuracy of confidence indicators is a means to assess their validity. As seen above, we can focus on the GDP as a measure of economic activity for a country. In the following, we compared the results obtained with the proposed strategy with the results obtained with the approaches of Taylor and McNabb (2007) and Gelper and Croux (2010). These approaches are based on the idea that there is a certain degree of co-movement between economic activity and sentiment. To this aim, the authors consider a vector auto-regressive (VAR) model:

9 $$\begin{aligned} {\mathbf{z}}_t = \alpha + \phi _1 {\mathbf{z}}_{t-1} + \phi _2 {\mathbf{z}}_{t-2} + \dots + \phi _p {\mathbf{z}}_{t-p} + {\overline{\varepsilon }}_t \end{aligned}$$

where ${\mathbf{z}}_{t-j} (j=0,\dots ,p)$ are the time vectors containing the GDP, the values of the confidence indicator and other covariates for the economic activity, with ${\overline{\varepsilon }}_t$ zero-mean serially uncorrelated error terms. Following previous studies, we choose the number of lags *p* of the model in Eq. (9) according to the Akaike Information Criterion (AIC).^7^ The parameters $\phi _j (j=1, \dots , p)$ are estimated using OLS. In this framework, Taylor and McNabb (2007) considered the GDP and quarterly samples covariates, while Gelper and Croux (2010) predicted the industrial production. As a consequence, Taylor and McNabb estimated the model with quarterly data, by temporally aggregating the confidence indicator, while Gelper and Croux considered predictions only on a short-term indicator.

We overcame the limitations of both approaches by considering the GDP as the economic activity indicator, and the ESI, the MF-CCI and $\text{MF-CCI}^{+}$ as composite confidence indicators that measure the economic sentiment. Moreover, we included industrial production and inflation rate as additional covariates. To avoid temporal aggregation of the high-frequency variables, we estimated a mixed-frequency VAR (Ghysels et al. 2016). Let consider that each vector ${\mathbf{z}}_t$ is formed by a low-frequency time series, defined as ${\mathbf{z}}^{LF}_t$ and other time series with higher frequency, defined as ${\mathbf{z}}^{HF}_t$. Ghysels et al. provided the following finite order VAR representation of stacked vectors:

10 $$\begin{aligned} \left[ \begin{array}{c} {\mathbf{z}}^{HF}_t\left( \tau _{L}, 1\right) \\ \vdots \\ {\mathbf{z}}^{HF}_t\left( \tau _{L}, m\right) \\ {\mathbf{z}}^{LF}_t\left( \tau _{L}\right) \end{array}\right] =A_{0}+\sum _{j=1}^{P} A_{j}\left[ \begin{array}{c} {\mathbf{z}}^{HF}_t\left( \tau _{L}-j, 1\right) \\ \vdots \\ {\mathbf{z}}^{HF}_t\left( \tau _{L}-j, m\right) \\ {\mathbf{z}}^{LF}_t\left( \tau _{L}-j\right) \end{array}\right] +{\overline{\varepsilon }}\left( \tau _{L}\right) \end{aligned}$$

where $A_0$ is a vector of constant, $A_j$ is matrix of parameters, $\tau _L$ is the low-frequency, *m* is the observed frequency mismatch and ${\overline{\varepsilon }}\left( \tau _{L}\right)$ is a vector containing both the low-frequency and high-frequency shocks. We use the model (10) to obtain short-term forecasts for the GDP. In the mixed-frequency VAR, we considered one low-frequency, i.e. the GDP growth rate, and three high-frequency variables, i.e. the growth rates of the sentiment indicators, the industrial production and the inflation, where the sentiment is computed in three different ways according to the aforementioned composite indicators. The time series of the GDP growth rates are shown in Fig. 1. The measures are represented as growth rates to ensure stationarity for the variables included in the mixed-frequency VAR model (10).

In order to evaluate the forecasting accuracy, we have split up the set into two equal-sized sub-sets: a *training* set used to estimate the parameters and obtain the forecasts, and a *testing* set used to evaluate the forecast accuracy. Given a composite confidence indicator *i*, to measure its predictive accuracy, we used a generic loss function $\ell _{i}$:

11 $$\begin{aligned} \ell _{it} = \ell (e_{it}) \quad \text{with} \quad e_{it} = {\hat{Y}}_{it} - Y_t \end{aligned}$$

where $Y_t$ is the vector of the actual values of the variables, ${\hat{Y}}_{it}$ is the vector of the forecasts obtained with the MF-VAR, and $e_{it}$ is the vector of the prediction error. About the choice of the loss function in Eq. (11), we considered a trimmed version of the Mean Square Forecast Error. Trimming is introduced to alleviate the effect of the COVID-19 outliers. As shown by previous papers (e.g., Armstrong and Collopy 1992; Chatfield 1992), we applied a 5% trim because it ensures an acceptable degree of robustness without a considerable cut in the sample size. Following Gelper and Croux (2010), we evaluated the over-performance of the proposed indicators by the ratio:

12 $$\begin{aligned} \text{ACC}_{newCCI} = \frac{\ell _{newCCI,t}}{\ell _{ESI,t}} \end{aligned}$$

We have an over-performance of the proposed indicator with respect to the ESI if $\text{ACC}_{newCCI} < 1$. The obtained ACC ratios are shown in Table 3.

**Table 3:** ACC ratios (with respect to the ESI) for the economic activity forecasts

	MF-CCI	$\textbf{MF-CCI}^{+}$
*GDP forecasts*		
Belgium	0.1129	0.1549
Germany	0.0720	0.0728
Greece	0.2908	0.2656
Spain	0.1224	0.1343
France	0.2834	0.3047
Italy	0.3256	0.3254

The results suggest that the mixed-frequency composite confidence indicators led to better forecasting performances for all the analysed countries. Table 3 shows the forecasting accuracy for GDP growth rates. The MF-CCIs indicators both dominated the ESI with ratios ever lower than 1. Moreover, the MF-CCI seemed to over-performs its extension for media sentiment ($\text{MF-CCI}^{+}$), except for Greece (with a better ACC ratio for $\text{MF-CCI}^{+}$) and Italy (where the ACC ratios are almost equal). This over-performance is mainly due to the direct approach in computing each component weights, by accounting for the possible business cycle fluctuations and the ability of each sector’s confidence to generate GDP fluctuations. Moreover, the inclusion of media sentiment in the $\text{MF-CCI}^{+}$ improved forecasts as well. With this respect, we have to recall the extensive empirical evidence documenting the important role of media sentiment, and the related concept of uncertainty intrinsic in the EPU, in determining fluctuations in the GDP. Indeed, this important information has been successfully considered in computing a broader confidence indicator. Nevertheless, the direct approach employed for weights’ computation seemed to have a more important role in improving the performances than the inclusion of additional information.

### Uncertainty and sensitivity analysis

The development of composite indicators has strengths and weaknesses. Among the advantages, we can mention that composite indicators facilitate the interpretation of complex phenomena, by synthesising many indices into one meaningful measure. Thus, composite indicators can assist decision-makers in their activities. Moreover, essential tasks such as cross-sectional analyses are easier because based on a single value instead of several characteristics. However, composite indicators have also some drawbacks. First, composite indicators require many data to be constructed, so it is an expensive process. Secondly, several choices have to be made to construct composite indicators, such as selecting the relevant dimensions, aligning the polarity of each sub-indicator, using a specific normalisation scheme for each sub-indicator, selecting the approach for their synthesis as well as the adopted weighting scheme. Therefore, constructing a composite indicator requires specific expertise. Furthermore, the ranks assigned to each case can be sensitive to any of the choices mentioned above. Most importantly, composite indicators can be misinterpreted. If this happens in a public context, policies could be oriented in the wrong direction. The misinterpretation of the policy messages highlighted by composite indicators is more and more possible when their construction is not robust (for a detailed discussion see Saisana et al. 2005).

A sensitivity analysis has to be used to assess the robustness of the constructed composite index. Considering all the above-mentioned aspects, the selection of sub-indicators, their normalisation and aggregation represent the most critical aspects. A critical aspect to evaluate in order to assess the robustness of a composite indicator is the inclusion/exclusion of the selected sub-indices, to evaluate their effect on the overall measure (Maggino 2017). Weighting is also essential, primarily when composite indicators are constructed using quantitative rather than qualitative approaches. It should be noted that, in studies regarding composite indicators, *min-max* and *z-score* are the most used normalisation approaches, but in a time series context, the min-max normalisation is usually preferred (e.g. see Alaimo and Maggino 2020; Alaimo et al. 2021; D’Urso et al. 2020; Mazziotta and Pareto 2018, 2016). Therefore, we evaluate the robustness of the proposed indicator for these risk factors by simultaneously using *uncertainty* (UA) and *sensitivity* (SA) analysis, as done in Saisana et al. (2005). The steps of the UA procedure can be summarised as follows:

- identify *K* risk factors $F_{k}(k=1, \ldots , K)$ that introduce uncertainty in the results: in our case we choose the normalization approach, the weighting scheme and the exclusion of indicators as the risk factors;
- assign a probability density function to each risk factor: our choices for PDF are shown in Table 4;
- generate randomly $L=1000$ combinations or samples of independent risk factors and, for each simulation, calculate the composite index for *n*-th each unit $c_{n,t}^{l}=f\left( x_{n,t,1}, x_{n,t,2}, \ldots , x_{n,t,I} ; F_{1}^{l}, F_{2}^{l}, \ldots , F_{K}^{l}\right) (n=1,2, \ldots ,N; l=1,2, \ldots , L; t=1,\dots ,T; i=1,\dots ,I)$, where $r_{n,t}^{l}={\text{rank}}\left( c_{n,t}^{l}\right)$ is the rank assigned by the composite index to unit *n* at time *t* for sample *l* and $r_{n,t}$ is the original rank of unit *n* at time *t*. Note that the function $f(\cdot )$ in our case is additive (see Sect. 3) so we include an additive structure among the risk factors as well;
- calculate the average rank of the country ${\bar{r}}_n = \frac{1}{T} \sum _{t=1}^{T} r_{n,t}$ for each country;
- calculate the average shift in the countries’ ranks:
  13 $$\begin{aligned} \bar{\text{R}}^{l}=\frac{1}{N} \sum _{n=1}^{N} \vert {\bar{r}}^l_n - {\bar{r}}_n \vert \end{aligned}$$
- analyse the main characteristics of the distribution of ${\bar{R}}^{l}$. The lower the variance, the greater the robustness.

**Table 4:** Sensitivity analysis: summary of the assumptions

Risk factor $F_k$	PDF	Detail
Normalisation	Uniform $U\sim [0,1]$	Min–Max if $U\le 0.5$
		Z-score if $U>0.5$
Weighting	Uniform $U\sim [0,1]$	Proposed weights if $U\le 0.5$
		Fixed weights if $U>0.5$
*Exclusion of indicator*		
Industrial	Discrete Uniform $U\sim \{1,2,3,4,5\}$	Exclusion if $U=1$
Services	Discrete Uniform $U\sim \{1,2,3,4,5\}$	Exclusion if $U=2$
Retail Trade	Discrete Uniform $U\sim \{1,2,3,4,5\}$	Exclusion if $U=3$
Construction	Discrete Uniform $U\sim \{1,2,3,4,5\}$	Exclusion if $U=4$
Consumer	Discrete Uniform $U\sim \{1,2,3,4,5\}$	Exclusion if $U=5$

**Fig. 4:**
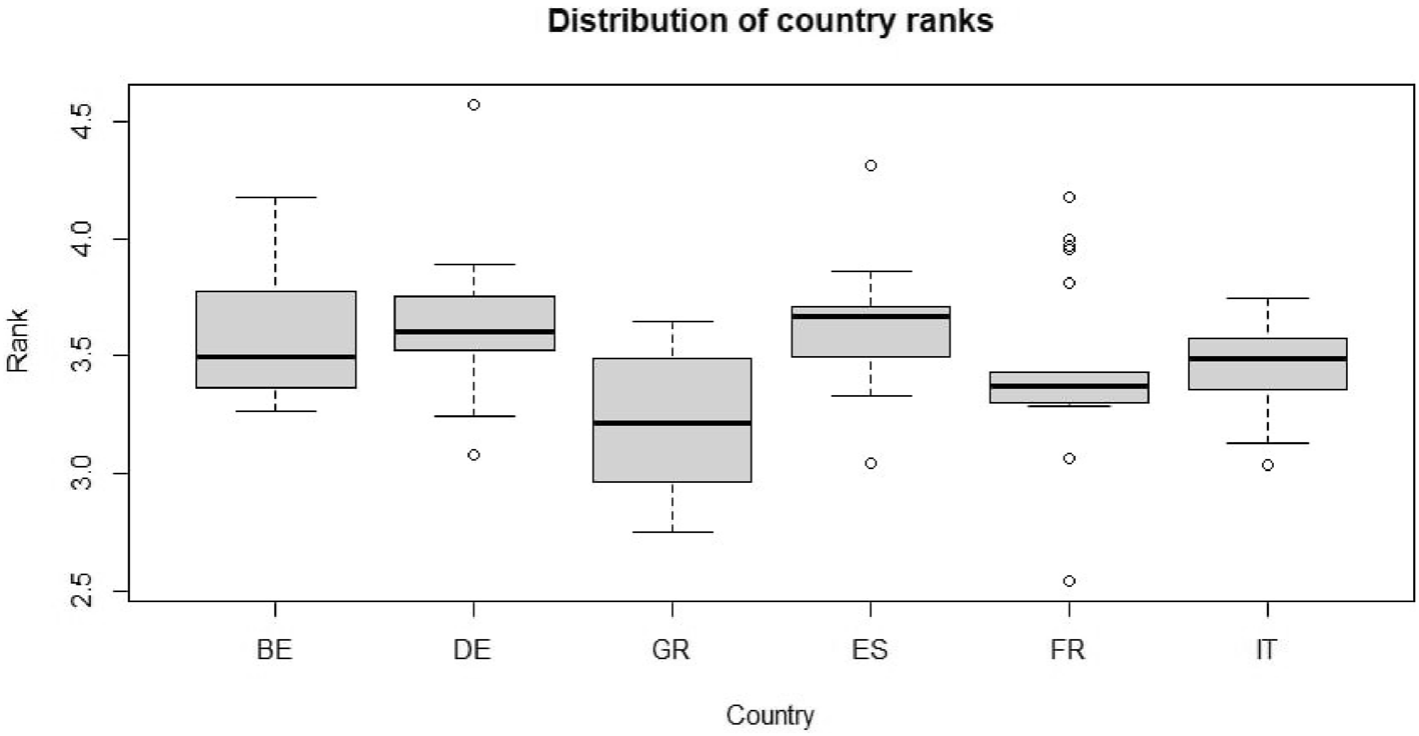
Distribution of country ranks ${\bar{r}}^{l}_n$ for *L* simulations

We compare the fixed weighting scheme adopted by the ESI (see Table 1) with the one proposed in the paper. When a single indicator is excluded, the fixed weights employed by the ESI are scaled to a unit sum. The EPU is not included in the sensitivity analysis to not introduce a distortion in the results. The country rank distributions obtained according to the *L* simulations, i.e. the distribution of ${\bar{r}}^l_n$, are shown in Fig. 4.

In terms of median rank, we observe that Spain is characterised by the highest level of sentiment, whereas Germany is second and Italy third. Then, Belgium and France have lower sentiment levels and are followed by Greece, the country with the lowest sentiment. Italy is the country with the most stable rank since its boxplot shows the lowest level of variability. In other words, the composite index is very robust for this country. On the contrary, France shows many outliers, and Greece is the country with the highest variability. However, Greece’s rank is lower than the medians of the other countries in the sample for most simulations. Similarly, Belgium is the only other country without outliers in the final ranking. Overall, the UA highlights that Germany, Spain and Italy have the highest and most stable ranks in terms of sentiment and that most of the countries’ ranks variability is induced by France and Greece.

Starting from the results of Uncertainty Analysis, we preform the Sensitivity Analysis in order to deeply analyse the differences in the average rank shifts ${\bar{R}}^l$. To this end, we adopt a variance-based technique, which is the most appropriate for conducting a sensitivity analysis in the context of composite indicators (Saltelli et al. 2000; Saltelli and Tarantola 2002).

We can measure the relevance of a risk factor $F_k$ with the so-called sensitivity index. For *k* independent input factors, the sensitivity index $S_k$ is defined as the fractional contribution to the model output variance $\text{V}$ due to the uncertainty in $F_{k}$, called $\text{V}_k$:

14 $$\begin{aligned} \text{S}_{k}=\frac{\text{V}_{k}}{\text{V}} \end{aligned}$$

where $\text{V}=\sum _k \text{V}_k$ is the output variance due to each $F_{k}$. Note that the formula of $\text{V}$ does not contain interactions, such that the total variance is equal to the sum of the single factors’ variances. A model of this type is called additive. Since the class of indicators considered in the paper is additive, we assume that the risk factors are additive, so that $\sum _k V_k =1$. In this case, the sensitivity index is given by (14).

The results of the sensitivity analysis are shown in Table 5.

**Table 5:** Sensitivity analysis: results

Risk factor	$S_k$
Normalisation	0.0979
Weighting	*0.2489*
Industrial exclusion	0.0829
Services exclusion	0.0830
Retail exclusion	*0.2854*
Construction exclusion	0.1097
Consumer exclusion	0.0921

In this case, we have that values greater than 0.14 indicate important input factors for the composition of the indicator. In other words, we consider an input factor important if $S_k>1/k$ (Saisana et al. 2005). The relevant factors, i.e. weighting approach and the exclusion of the Retail trade indicator, are highlighted with italics font in Table 5. The sensitivity analysis highlights that the uncertainty induced by the selection of the normalisation scheme is negligible for the construction of the composite indicator. In other words, the countries’ ranks are robust for the choice of normalisation. Furthermore, the exclusion of industrial or consumer confidence seems irrelevant in terms of final countries’ ranks, whereas most of the variability is induced by excluding the retail trade sub-indicator. Note that the ESI assigns a lighter weight to this indicator, which is very important for the definition of country ranks. Then, we observe that the weighting scheme is, to some extent, important in terms of country ranks’ robustness, even if its relevance is much smaller than the exclusion of indicators. However, we can argue that selecting an accurate weighting scheme is crucial. With this respect, we have already mentioned the limits associated with the ESI weighting scheme. Among the other schemes, previous studies highlight that the ESI’s fixed weighting scheme does not take into account the fluctuations in the business cycle and the relationship between confidence and GDP. Therefore, this fixed weighting scheme may be potentially meaningless from an economic point of view. Hence, as we argued in previous sections, the proposed weighting approach should be used instead of the fixed weights employed by the ESI.

## Concluding remarks and policy implications

In an increasingly hectic and complicated world such as the one we live in, policymakers need a more significant amount of data of different nature and greater timeliness to support their decision processes. The orientation of public opinion, in particular, is an element more and more considered by governments as well as by scholars and researchers interested in economic forecasting. How pessimistically or optimistically people and firms view the economy has both economic and political consequences. Perceptions about present economic conditions and expectations of future changes represent fundamental factors of attitude formation and, as a consequence, of political behaviour. Some authors (e.g., Schneider 1984; Zagórski and McDonnell 1995) showed that public sentiment is a good predictor of voting preferences. Voters participating in general elections evaluate the governments by their performance over the legislative period, but their preferences are determined much more strongly by expectations of changes in economic conditions. The implications for the governments of a negative or positive sentiment rely on their choices in monetary or fiscal policies in a domestic dimension as well as on their decisions about foreign affairs. Caldara and Iacoviello (2008) stated that the geopolitical risks affect financial markets and business cycles, and recently Pehlivanoğlu et al. (2021) analysed the effects of the resulting uncertainty on consumer and business sentiment. Confidence indicators offer valuable insights into this sentiment.

This paper proposes two new composite confidence indicators (MF-CCI and $\text{MF-CCI}^{+}$), based on mixed-frequency data, with the aim of better measuring the evolution over time of public sentiment. The idea behind these indicators is that, differently from ESI calculated by the EU, weights associated with each component are not proportional to the share of produced GDP. Indeed, the MF-CCIs consider the impact of each confidence component on the GDP in a dynamic perspective. The more significant is the change in GDP generated by a change in the sentiment of an economic sector, the greater is the weight assigned to the sector itself. An additional advantage of the MF-CCIs indicators is that they do not need temporal disaggregations, as in Sorić et al. (2016) or aggregations, as in Claveria et al. (2018), to be constructed. All the high-frequency fluctuations within each low-frequency period are used to build the composite confidence indicator without any loss of information. In the case of the $\text{MF-CCI}^{+}$, we also considered an expanded information set by including a proxy for media sentiment. Several authors showed that the role of media is crucial in orienting firm opinion, and the media sentiment itself can be used as a leading indicator in a forecasting strategy (e.g., Seki et al. 2022).

To test the effectiveness of our two novel indicators, we studied the sentiment reaction of households and firms to the restrictions introduced by local governments to reduce the COVID-19 pandemic spread in a sample of EU economies, including both virtuous countries and PIIGS countries. We showed that the MF-CCI movements are more likely to explain fluctuations in sentiment rather than the ESI. Hence, the proposed approach seems to provide a more reliable measure of public opinion. We also found that the sentiment decreased after introducing restrictive measures, as was expected. These adverse effects on sentiment levels were much lower in the second wave than in the first one. Since composite confidence indicators are nowadays commonly used in forecasting problems, we evaluated, in addition, the MF-CCIs’ forecasting accuracy. With this aim, we performed a forecasting analysis to understand which confidence indicator, among the two MF-CCIs and the ESI, can be more appropriate in predicting economic activity. As economic activity measure, we considered the GDP growth rate. Because of frequency mismatch, we employed a mixed-frequency VAR model for obtaining the predictions. The overall findings suggested that using the MF-CCIs indicators as predictors for economic activity significantly improves the accuracy—in terms of loss function—with respect to the ESI. This latter means that the proposed measures may be used as relevant leading indicators and can successfully predict economic activity.

A relevant conclusion drawn from the case study here presented is that the impact of shocking events on public sentiment should not be underestimated. As recently discussed by Stiglitz (2021), a dispersion of beliefs among economic agents increases the macroeconomic volatility of a country, and it may lead to severe economic unbalances and downturns. This aspect is particularly important when the state of crisis induced by the shocks is not foreseeable in a short time, like the concurrent social and economic emergency induced by the COVID-19 pandemic. In this sense, an indicator such as the MF-CCI may help policymakers prevent a decline in people’s and firms’ sentiment and have near-real-time evidence of the effect of the implemented measures on public opinion, preventing macroeconomic fluctuations. A similar advantage can also be gained by encompassing the media sentiment in the confidence measure, as in the $\text{MF-CCI}^{+}$. The critical reading of the economic phenomena given by newspapers and news agencies could offer an alternative but concurrent viewpoint that have to be taken into account by policymakers and politicians at large, primarily because the opinions of media often reflect the official and unofficial positions of relevant interest groups and intermediary bodies (Binderkrantz et al. 2017; Dür 2019; Eachempati and Srivastava 2022). On the other hand, as stressed above, the use of confidence indicators as significant leading indicators in GDP predictions has been recently debated by scholars and practitioners, highlighting how this kind of data could be employed within everyday economic analysis in a “nowcasting” perspective. Since most macroeconomic variables are feasible only after some lag, having meaningful up-to-date indicators of the state of the economy becomes a priority. Our findings may serve as a reference for governments, central banks, and research institutes interested in promptly predicting the economic health of a country or a group of countries (as the Euro Area, for example), offering a tool for making informed economic and policy decisions in a narrower time window than the usual quarterly-based horizon.

The proposed approach has been tailored starting from the ESI, therefore it is possible to calculate the MF-CCIs for all the EU countries and some other countries participating in the Joint Harmonised EU Programme of Business and Consumer Surveys as EU candidate members (e.g., Albania and Turkey). Nevertheless, the debate about the use of sentiment surveys in economic analyses interested during the past years also other non-EU economies. Moon (2011), for example, proposed the construction of an Economic Sentiment Indicator for the Korean Economy. Kitrar and Lipkind (2021), discussed the relationship between sentiment and GDP growth in Russia—mainly focusing on COVID-19 period—showing how a composite confidence indicator can be effectively used as a leading indicator in forecasting analyses. In a similar fashion, other authors focused on the use of business confidence only as additional leading, like Ginker and Suhoy (2022) for Israel, or the use of both business confidence and consumer confidence, like Kabundi et al. (2016) for South Africa. This means that the MF-CCIs’ logic can be extended to other countries outside the EU when an informative set comparable with ESI is available. Clearly, the differences in data collection and the construction of the sub-indicators have to be considered, especially in comparing EU and non-EU economies.

As a further development of the MF-CCIs, other measures of the media sentiment can be tested, for example, by considering the text mining strategies discussed in the literature review. A textual-based confidence indicator may be constructed by considering the official social media accounts (e.g., Twitter) of the prominent newspapers and news agencies at a country level, avoiding the social hacking problem. The construction of indicators based on new kinds of data as textual data is an open topic in the economic and social literature, offering an alternative insight not possible with more traditional data. Moreover, a different view of consumer and business expectations may be used in the evaluation of public sentiment, taking into account the movements of the different stances as in the Economic Climate Tracer introduced by DG ECFIN in last years to analyse EU business cycle (Gayer and Genet 2006).

## Footnotes

1 Alongside the paper, the terms *confidence* and *sentiment* used to define the quantitative measure of public opinion about the economy are considered interchangeable.

2 Detailed information about the legislative acts about COVID-19 in Italy can be found at the following link https://www.openpolis.it/coronavirus-lelenco-completo-degli-atti.

3 PIIGS is an acronym used to identify Portugal, Italy, Ireland, Greece, and Spain, the weakest economies in the Euro Area during the sovereign debt crisis began at the end of 2009.

4 Data about GDP have been retrieved from https://ec.europa.eu/eurostat/web/main/data/database in the section related to national accounts. Confidence surveys data have been downloaded from https://ec.europa.eu/info/business-economy-euro/indicators-statistics. EPU data have been downloaded from https://www.policyuncertainty.com.

5 We employed the packages midasr for the temporal alignment and quadprog for the implementation of the constrained mixed-frequency optimisation problem in (6). The R function used for the construction of the mixed frequency composite indicator is available under request.

6 https://www.europarl.europa.eu/RegData/etudes/STUD/2021/662903/IPOL_STU(2021)662903_EN.pdf.

7 The AIC is one of the most employed criteria for choosing the optimal number of lags in VAR models. See, for example, Ozcicek and McMillin (1999).
